# Assessment of the frequency of sleep complaints and menopausal symptoms in climacteric women using the Jenkins Sleep Scale

**DOI:** 10.5935/1984-0063.20200041

**Published:** 2021

**Authors:** Alvaro Monterrosa Castro, Teresa Beltrán-Barrios, María Mercado-Lara

**Affiliations:** Grupo de Investigación Salud de la Mujer. Universidad de Cartagena, Facultad de Medicina - Cartagena - Bolivar - Colombia.

**Keywords:** Climacteric, Menopause, Sleep, Adjustment Disorders, Depression

## Abstract

**Objective:**

To identify the frequency of sleep complaints (SC) and associated menopausal symptoms in climacteric women, apparently healthy, residing in three different capital cities of the Colombian Caribbean.

**Material and Methods:**

Cross-sectional study which is part of the investigation project CAVIMEC [*Calidad de Vida en la Menopausia y Etnias Colombianas*]. Data were collected by interviewers, on a door-to-door visit. Healthy women residing in the Colombian Caribbean, 40-59 years old, were studied. Sociodemographic characteristics form and scales were applied: Menopause Rating Scale, Jenkins Sleep Scale, Perceived Psychological Stress (perceived stress), Goldberg Anxiety and Depression Scale, SCOFF scale (eating disorders), and Loneliness Scale by Hughes. The women were divided into two groups: with SC and without SC, according to the Jenkins scale result. Crude and adjusted logistic regressions were performed: SC (dependent variable) with sociodemographic characteristics and the results of the scales used (independent variables).

**Results:**

Five hundred eighty-ﬁve women were studied. 16.5% with SC. No differences were observed in age, BMI, and high blood pressure. Proportionally more women with SC had depression, anxiety, perception of loneliness, severe menopausal symptoms, somatic, psychological, urogenital, and quality of life severe impairment (*p*<0.05). There were no differences in eating disorders and perceived stress. In the adjusted model, only depression was associated with SC, OR: 9.81 [95% CI: 1.29-74.3], p<0.05.

**Conclusion:**

SC were identiﬁed in 16.5% of the climacteric women of the Colombian Caribbean. In an adjusted model, probable depression was the only factor associated with SC.

## INTRODUCTION

Sleep is a vital physiological process that allows the proper functioning of the immune system, metabolic, and cognition^1^^,^^2^^,^^3^. Sleep rhythm can be altered by endogenous disruption of the sleep-wake cycle or by biopsychosocial influences^4^. Sleep disturbances are usually grouped under the term sleep disorders and can generate physiological and biological impairment^1^^,^^2^^,^^5^^,^^6^.

Sleep disorders are present in both men and women; however, the risk of developing them is double in females^7^^,^^8^. Sleep complaints (SC) are part of menopausal symptoms and their frequency varies according to ethnic groups, culture, socioeconomic levels, or geographical areas that negatively impact health resources and the climacteric, a vital stage of the woman in which disappears the reproductive function by endocrinological mechanisms^1^^,^^2^^,^^4^^,^^6^^,^^9^.

Medical attention during climacteric should be multidisciplinary, it should provide guidance and timely assistance to reduce symptoms severity and promote the empowerment of the women against them^5^^,^^6^^,^^10^. Traditionally, the clinical approach has focused more on hot flashes, leaving aside SC, which are also negatively associated with quality of life and health conditions^5^^,^^6^^,^^11^^,^^12^.

Midlife women from different Latin American regions, with different ethnicities, have their habits, traditions, and cultural norms, which can modify the frequency of menopausal symptoms^4^^,^^13^^,^^14^. Data on SC are limited in these population groups. Furthermore, most studies of SC have been performed in patients or in hospital settings. More information about SC is needed, especially in women in climacteric who have not considered it necessary to consult the health professional for SC. The objective was to identify the frequency of SC and associated menopausal symptoms in climacteric women, apparently healthy, residing in three different capital cities of the Colombian Caribbean.

## MATERIAL AND METHODS

### Study design

Cross-sectional study which is part of the investigation project CAVIMEC [*Calidad de Vida en la Menopausia y Etnias Colombianas*]. Data was collected in 2019 by interviewers with training in social promotion, education, or nursing assistants, who made door-to-door visits in residential neighborhoods of medium socioeconomic status, in three cities located at sea level: Cartagena, Barranquilla, and Monteria in the Colombian Caribbean. The pollsters came to each dwelling, questioned about the presence of women who met the study criteria and invited them to participate. They explained in detail the scope of the investigation, arranging the moment for the application of the form. During the surveys, the tools to be used and the anonymous and voluntary nature of the study were explained; the written consent was requested, and anthropometric measures were carried out. After that, the surveys were reviewed: those incomplete were classified as “deleted” and those that were correctly filled out as “accepted”.

### Participants

The participants were healthy women, between 40-59 years old, who carried out their daily activities at home or work. Each of them defined the ethnic group to which they belonged by self-recognition and according to their racial phenotypic characteristics. Pregnant women, those with diagnosed or treated mental or neuromotor illnesses, with reading and writing disabilities and illiterate were excluded from the form. Women with a history of consultations or treatments for sleeping sickness were also excluded, to search for SC in healthy women in general and not in patients. It should be noted that the participants could withdraw at any time.

### Tools

A form with three parts was designed. The first part evaluated sociodemographic variables (age, ethnic group, studies, work activity, marital status, children, smoking habit, drinking coffee, wine, beer or liquor, and sexual partner). The use of hormonal therapy and a history of hysterectomy were questioned. The stages of the climacteric life cycle were defined according to Stages of Reproductive Aging Workshop (STRAW) criteria + 10^15^ as menopausal transition early (cycles with less than seven days of variation), menopausal transition late (irregular cycles with more than seven days of variation or repeated absence of bleeding) and postmenopausal (menstrual absence greater than twelve months).

The second part had seven scales. Initially, the Jenkins Sleep Scale (JSS) was applied to explore the presence of SC in the last month. JSS is composed of four items: difficulty falling asleep, waking up several times per night, difficulty staying asleep or waking far too early, and waking up exhausted the next morning after the usual amount of sleep. Points are assigned for each item as follows: zero (never), one (1-3 days), two (4-7 days), three (8-14 days), four (15-21 days) and five (22-28 days). The total score of JSS ranges from 0 to 20, with higher scores indicating more SC. Women with a total JSS score from 1 to 11 were defined as having infrequent sleep problems (not SC or without SC) and those with a score ≥12 were defined as having frequent sleep problems (yes SC or with SC)^16^^-^^18^. The Menopause Rating Scale (MRS) was used to identify the presence and severity of the menopausal symptoms. The MRS is a specific quality of life scale in menopause, made up of eleven questions grouped into three dimensions: somatic, psychological, and urogenital. The higher the score, the worse the assessment of the quality of life, domains, and symptoms. A score >8 in somatic, >6 psychological, >3 urogenital, and >16 in quality of life was defined as a severe impairment of the respective dimension^19^.

The other scales were carried out to identify four biopsychosocial situations. The Perceived Stress Scale (PSS-14) identifies stress situations in the last month: the higher the score, the worse the assessment^20^. The Goldberg Anxiety and Depression Scale (GADS) has two subscales and defines the presence of probable anxiety (four or more affirmative questions) and probable depression (two or more affirmative questions)^21^. The SCOFF Scale, whose name derives from an acronym for the initials of each item (Sick, Control, Out weight, Fat, Food), assesses symptoms related to eating behavior in the last three months; two or more points indicate probable eating disorder^22^. The Hughes Loneliness Scale that, employing three questions, considers the perception of loneliness and it has no cutoff point^23^. For the present study, the presence of loneliness was estimated as the above-average value of the total score on the Hughes Loneliness Scale.

In the third part, anthropometric measurements were recorded: body weight in kg and height in cm, as well as an abdominal circumference at the umbilical level and hip circumference at the iliac crests. The body mass index was calculated by dividing the weight by the squared height, according to the criteria of the World Health Organization^24^. Abdominal circumference greater than 88cm was considered obesity according to the ATP-III criteria^25^.

### Sample size

The *Departamento Administrativo Nacional de Estadísticas* (DANE) of Colombia^26^, according to the projection of the Colombian population census of 2005, estimated 1.405.602 women residing in Cartagena, Barranquilla, and Monteria in the Colombian Caribbean in 2018, of whom 40% resided in the first of the mentioned cities, 45% in the second, and 15% in the third. Of these, 333.957 were between 40-59 years (23.7%), which are the universe of the study. A total of 541 participants were estimated to be included in the study using the online calculator, *Netquest*^©^, considering the sample size with 50% heterogeneity, 98% reliability, and a 5% margin of error. Five hundred and ninety-five forms were applied, after adding 54 (10%) to compensate for incomplete forms, which were stratified between the three cities according to population sizes.

### Statistical analysis

The accepted forms were typed into a database with *Microsoft Excel*^©^. Statistical analysis was performed with SPSS-21.0^©^. Two groups were formed according to the SC evaluated with the JJS: group A, women without SC (score 1-11), and group B, women with SC (score equal to or greater than 12). Results of JJS are presented as means and standard deviation. Continuous data are expressed in median (Me) and interquartile range (IR), by non-parametric distribution according to Q-Q graphs and Kolmogorov-Smirnov normality test. Categorical data are expressed in absolute values, percentages, and 95% CI. The differences of the medians were evaluated with the Mann-Whitney U test o Anova and the percentages with the chi-square o Fisher exact test. Cronbach’s alpha was calculated for the JJS. Logistic regression was performed to estimate the association of SC (dependent variable) with menopausal symptoms, deterioration in the quality of life, biopsychosocial situations, and sociodemographic characteristics (independent variables). Crude and adjusted odds ratio (age, ethnicity, work activity, marital status, sexual partner, wine consumption, hormonal therapy, climacteric state, urinary incontinence, probable anxiety, probable depression, loneliness, quality of life, and the eleven menopausal symptoms that MRS evaluates), as well as the 95% CI. For all comparisons *p*<0.05 was considered statistically significant.

### Ethical aspects

The participants signed the written consent before completing the form following the Declaration of Helsinki’s principles. Scientific, technical, and administrative standards were taken into account for health research, established in resolution No. 8430-1993 of the Ministry of Health of the Republic of Colombia, which allows this study to be considered as minimal risk research^27^. The CAVIMEC research project is approved by the Ethics Committee of the Universidad de Cartagena, Colombia.

## RESULTS

Of 595 forms applied, 10 were incomplete (1.6%); therefore, 585 women were included in the study, 44 (8.1%) more than what was established in the sample size. The mean age was 47.0 (7.5) years. More than 90% recognized themselves as mestizo, 60% carried out housewife activities and nine out of ten lived with their sexual partner. Less than 8.0% had diabetes. Very few of them consumed wine, beer, liquor, or were current smokers, <2.0%. Half were postmenopausal and 30% had been hysterectomized.

Table 1 presents the sociodemographic characteristics of this population, as well as the distribution in accordance with the SC presence: 488 (83.4%) did not present it, but it was present in 97 of them (16.5%). No significant differences were observed between the groups, except in years of study and probable depression. Half of the women in each group were postmenopausal (*p*=0.35).

**Table 1:** Sociodemographic characteristics.

	All n=585	Without sleep complaint n=488 (83.4%)	Withsleep complaint n=97 (16.5%)	p
		Me (RI)		
Age	47.0 [7.0]	47.0 [7.0]	47.0 [8.0]	0.15^§^
Years of studies	11.0 [1.0]	11.0 [1.0]	11.0 [1.0]	0.03*
Body mass index	27.3 [6.8]	27.2 [6.9]	27.9 [6.2]	0.08^§^
Waist/hip ratio	0.7 [0.2]	0.7 [0.1]	0.7 [0.1]	0.45^§^
Daily cups of coffee	1.0 [1.0]	1.0 [1.0]	1.0 [1.0]	0.17*
		**% (95% CI)**		
Normal nutritional status	30.6 [26.9-34.5]	31.7 [27.7-36.0]	24.7 [16.5-34.5]	0.49***
Under weight	0.5 [0.1-1.6]	0.6 [0.2-1.7]	0.0 [0.0-0.0]	
Overweight	38.6 [34.7-42.7]	38.1 [33.9-42.5]	41.3 [31.3-51.6]	
Obesity	30.3 [26.6-34.2]	29.5 [25.6-33.7]	34.0 [24.7-44.3]	
Mixed ethnicity	92.7 [90.2-94.6]	92.2 [89.4-94.2]	94.8 [88.3-98.3]	0.56***
Afro-descendant	3.9 [2.6-5.9]	4.3 [2.8-6.4]	2.1 [02-7.2]	
Indigenous	3.4 [2.2-5.3]	3.4 [2.1-5.5]	3.1 [0.6-8.7]	
Working labor activity	12.8 [10.3-15.9]	12.3 [9.6-15.5]	15.4 [8.9-24.2]	0.20***
Housewife	59.8 [55.7-63.8]	59.8 [55.4-64.0]	59.7 [49.3-69.6]	
Office worker	9.4 [7.2-12.1]	10.0 [7.6-13.0]	6.1 [2.3-12.9]	
Trader	12.7 [10.1-15.7]	12.3 [9.6-15.5]	14.4 [8.1-23.0]	
Retired	1.4 [0.6-2.8]	1.0 [0.4-2.3]	3.0 [0.6-8.7]	
Professional	3.9 [2.6-5.9]	4.5 [3.0-6.7]	1.0 [0.0-5.6]	
Single	6.2 [4.4-8.5]	6.5 [4.6-9.1]	4.1 [1.1-10.2]	0.70***
Married	23.1 [19.8-26.8]	22.1 [18.6-26.0]	90.4 [84.1-94.8]	
Common-law marriage	67.0 [63.0-70.8]	67.4 [63.1-71.4]	64.9 [54.5-74.3]	
Divorced	2.7 [1.6-4.5]	2.8 [1.7-4.7]	2.0 [0.2-7.2]	
Widow	1.0 [0.4-2.3]	1.0 [0.4-2.3]	1.0 [0.0-5.6]	
Menopausal transition early	27.0 [23.5-30.8]	26.0 [22.3-30.0]	31.9 [22.8-42.2]	0.35***
Menopausal transition late	17.1 [14.2-20.5]	16.8 [13.7-20.3]	18.5 [11.3-27.7]	
Postmenopausal	55.9 [51.8-60.0]	57.1 [52.7-61.4	49.4 [39.1-59.8]	
Diabetes	7.8 [5.9-10.3]	7.5 [5.5-10.2]	9.2 [4.3-16.8]	0.57**
Arterial hypertension	23.5 [20.3-27.2]	22.3 [18.8-26.2]	29.9 [21.0-40.0]	0.10**
Sexual partner	93.1 [90.8-94.9]	93.2 [90.6-95.1]	92.7 [85.7-97.7]	0.87**
Daily coffee consumption	83.8 [80.5-86.6]	84.2 [80.7-87.1]	81.4 [72.2-88.6]	0.49**
Monthly wine consumption	1.0 [0.4-2.3]	1.0 [0.4-2.3]	1.0 [0.0-5.6]	1.00***
Monthly beer consumption	2.7 [1.6-4.5]	3.0 [1.8-5.0]	1.0 [0.0-5.0]	0.26**
Monthly liquor consumption	0.7 [0.2-1.9]	0.6 [0.2-1.7]	1.0 [0.0-5.6]	0.64**
Never smokers	88.0 [85.1-90.5]	88.3 [85.1-90.8]	86.6 [78.1-92.6]	0.78**
Hormone therapy use	7.5 [5.6-9.9]	7.3 [5.3-10.0]	8.2 [3.6-15.6]	0.76**
Previous hysterectomy	32.8 [29.1-36.8]	32.1 [28.1-36.4]	36.0 [26.5-46.4]	0.45**
Probable anxiety	83.0 [79.8-85.9]	82.1 [78.5-85.3]	87.6 [79.3-93.4]	0.19**
Probable depression	90.6 [87.9-92.7]	88.9 [85.8-91.4]	98.9 [94.3-99.9]	<0.001**
Probable eating disorder	20.3 [17.2-23.9]	19.6 [16.3-23.4]	23.7 [15.6-33.4]	0.36**
Loneliness perception	57.7 [43.7-61.7]	57.1 [52.7-61.4]	60.8 [50.3-70.5]	0.50**
Pc	75.5 [71.9-78.8]	74.3 [70.3-78.0]	81.4 [72.2-88.6]	0.13**

§ Anova;

* Mann-Whitney U test;

** Chi-square - Mantel-Haenszel;

*** Fischer exact test.

In the last month, less than 20% of the studied participants never presented the four items evaluated by the JJS. In turn, less than 10.0% presented them for more than fifteen days. Four out of ten had difficulty falling asleep, difficulty staying asleep, or woke up several times per night for one to three days in the month before the application of the forms. Cronbach’s α was obtained: 0.825. The data obtained with the JJS are presented in Table 2.

**Table 2:** Jenkins Sleep Scale (n=585).

How often in thepast month didyou?	Never	1-3 days	4-7 days	8-14 days	15-21 days	22-28 days
			% (95% CI)			
Difficulty fallingasleep	18.9 [16.0-22.3]	36.2 [32.4-40.2]	21.5 [18.4-25.0]	14.5 [11.9-17.6]	3.9 [2.6-5.8]	4.7 [3.3-6.8]
Waking up several timesper night	17.4 [14.5-20.7]	37.0 [33.2-41.0]	25.6 [22.2-29.3]	10.6 [8.3-13.3]	4.7 [3.3-6.8]	4.4 [3.0-6.4]
Difficulty staying asleep or waking far tooearly	12.6 [10.2-15.5]	32.8 [29.1-36.7]	29.4 [25.8-33.2]	15.9 [13.1-19.0]	4.7 [3.3-6.8]	4.4 [3.0-6.4]
Waking up exhausted the next morning after usual amount of sleep	14.7 [12.0-17.8]	28.8 [25.3-32.6]	167(28.5) [25.0-32.6]	18.4 [15.5-21.8]	6.5 [4.7-8.7]	2.9 [1.8-4.6]

The following were the scores found for each item on the JJS, ordered from highest to lowest: waking up exhausted the next morning after the usual amount of sleep 1.81±1.24, difficulty staying asleep or waking far too early 1.80±1.24; difficulty falling asleep 1.62±1.31, and waking up several times per night 1.61±1.27. There were differences in all the scores of the four items when comparing the groups, being greater in those with SC (<0.001). The total score was 6.86±4.15. Women without SC had 5.50±2.94 and those with SC 13.73±2.01 (<0.001).

Menopausal symptoms assessed with MRS, except vaginal dryness, were found to be more frequent in women with SC. However, the differences were significant only in cardiac disturbances, sleep disorders, physical and mental fatigue, and muscle/joint discomfort. In turn, among the women with SC, a greater presence of severe impaired somatic, psychological, urogenital, and quality of life was observed (Table 3).

**Table 3:** Severe menopausal symptoms and severe impairment of quality of life Menopause Rating Scale.

	Alln=585	Without sleep complaint n=488 (83.4%)	Withsleep complaint n=97 (16.5%)	p
		% (95% CI)		
**Severe menopausalsymptoms**				
Hot flashes	12.1[9.7-15.0]	11.6 [9.1-14.8]	14.3 [8.1-23.0]	0.44*
Cardiac disturbances	8.3 [6.3-10.9]	7.1 [5.2-9.8]	14.4 [8.1-23.0]	<0.05*
Sleep disorders	13.5 [10.9-16.5]	12.0 [9.4-15.2]	20.6 [13.0-30.0]	<0.05*
Depressed mood	15.2 [12.5-18.3]	14.1 [11.3-15.5]	20.6 [13.0-30.0]	0.10*
Irritability	15.5 [12.8-18.7]	14.5 [11.7-17.9]	20.6 [13.0-30.3]	0.13*
Anxiety	15.7 [13.0-18.9]	15.6 [12.2-18.6]	18.5 [11.3-27.7]	0.40*
Physical and mental fatigue	20.3 [17.2-23.7]	17.6 [14.5-21.2]	34.0 [24.7-44.3]	<0.001*
Sexual disturbances	10.6 [8.3-13.3]	10.2 [7.8-13.2]	12.3[6.5-20-6]	0.53*
Bladder disturbances	9.0 [6.9-11.6]	8.4 [6.2-11.2]	12.3 [6.5-20.6]	0.21*
Vaginal dryness	10.7 [8.5-13.5]	11.0 [8.5-14.1]	9.2 [4.3-16.8]	0.60*
Muscle/joint discomfort	10.4 [8.2-13.1]	9.0 [6.7-11.8]	17.5 [10.5-26.5]	<0.05*
**Severeimpairment**				
Somatic	18.0 [15.0-21.4]	17.2 [14.1-20.8]	21.6 [13.9-31.1]	0.29*
Psychological	52.1 [48.0-56.2]	49.1 [44.7-53.6]	67.0 [56.7-76.2]	<0.001*
Urogenital	66.3 [62.3-70.1]	64.5 [60.2-68.6]	75.2 [65.4-83.4]	<0.05*
Quality of life	57.1 [53.0-61.1]	54.3 [49.8-58.6]	71.1 [61.0-79.8]	<0.001*

* Chi-square -Mantel-Haenszel.

In the analysis with unadjusted logistic regression, the symptoms that were significantly associated with a higher presence of SC were observed (Table 4). None of the sociodemographic characteristics, nor probable anxiety, perceived stress, a probable eating disorder, or perception of loneliness, were significantly associated. In the adjusted model, probable depression identified by GADS was the only significantly associated variable.

**Table 4:** Factors associated with sleep complaints - logistic regression (n=585).

Variable	Unadjusted		Adjusted	
	OR(95% CI)	p	OR (95% CI)	p
Probable depression^(*)^	11.94[1.6-87.40]	<0.05	9.81[1.29-74.3]	<0.05
Severe physical and mental fatigue^(**)^	2.41 [1.41-3.80]	<0.001	1.68[0.95-2.95]	0.07
Severe cardiac disturbances^(**)^	2.18 [1.12-4.23]	<0.05	0.99 [0.45-2.20]	0.99
Severe muscle/joint discomfort^(**)^	2.14 [1.16-3.93]	<0.05	1.54 [0.80-2.97]	0.18
Severe psychological impairment^(§)^	2.09 [1.32-3.35]	<0.001	1.18 [0.56-2.47]	0.64
Severe quality of life impairment^(***)^	2.07 [1.29-3.39]	<0.001	1.48 [0.57-3.88]	0.41
Severe sleep disturbances^(**)^	1.88 [1.07-3.31]	<0.05	1.25 [0.68-2.30]	0.46
Severe urogenital impairment^(§)^	1.66 [1.01-2.74]	<0.05	0.69 [0.31-1.54]	0.36

(*) Symptom evaluated with The Goldberg Anxiety and DepressionScale;

(**) Symptom evaluated with Menopause RattingScale;

(***) Assessed with the total score of the Menopause RatingScale;

(§) Assessed with the Menopause Rating Scale Domain.

## DISCUSSION

With the JJS, the prevalence of SC in climacterics in the Colombian Caribbean was much lower than the 37.5% reported by Ornat et al.^17^. Using the same scale, they evaluated 288 Spanish women aged 40-59 years, attending gynecological evaluation in an outpatient center. The results of frequencies obtained in clinical settings will be different from those obtained in communities since the former assess patients who in one way or another come to the clinic and the latter explores the opinions of women.

Monterrosa et al.^13^ reported that 57.1% of 1078 Colombian Caribbean and Pacific women had poor sleep quality when evaluated with the Pittsburgh Sleep Quality Index. Blümel et al.^4^ identified in 6079 Latin American climacteric women that half of them had insomnia, poor sleep quality, or both when evaluated with the Athens Insomnia Scale and the Pittsburgh Sleep Quality Index. The Study of Women’s Health Across the Nation (SWAN)^28^ found that 38% of women aged 40-55 years reported sleep disorders related to menopause when asked if they had difficulty sleeping in both previous weeks. Martínez^9^ found in a population of Cuban climacteric women who consulted for SC that 31.9% had problems initiating sleep or difficulty maintaining it. The differences in these figures are explained by the different tools used, as well as the sleep disorders evaluated.

As the climacteric state progresses, the frequency of SC is modified^4^^,^^5^^,^^13^^,^^29^. In the present study, 19% of premenopausal women, 18% of those who were in transition to menopause, and 15% of postmenopausal women reported SC. SWAN^30^ also reported that the prevalence of SC increases in climacteric: 16-42% premenopausal, 39-47% perimenopausal, and 35-60% postmenopausal. Joffe et al.^10^ indicated that women report SC more frequently as it passes from the late reproductive stage to perimenopause, which is also asserted by other authors^4^^,^^28^^,^^31^. This is explained by the modifications in the noradrenergic, serotonergic, GABAergic, and dopaminergic systems, as a consequence of age and hormonal changes^1^^,^^2^^,^^4^^,^^7^. Complete depletion of brain serotonin, in animal models, has been reported to lead to insomnia^2^^,^^7^^,^^32^.

The interactions between neurotransmitters and neurons are complex and participate in the sleep-wake balance^1^^,^^7^^,^^32^. Melatonin, serotonin, glycine, acetylcholine, adenosine, and gamma-aminobutyric acid are sleep inducers, while epinephrine, norepinephrine, histamine, orexin, and dopamine maintain wakefulness^2^. Two interconnected cycles have been described: homeostatic and circadian. The desire to sleep is automatically controlled by the former and depends on the amount of previous wakefulness; the latter is regulated by an endogenous pacemaker, located in the suprachiasmatic nucleus of the hypothalamus^1^. Over the years, both are altered by hormonal or metabolic influences, expressing themselves clinically with changes in the sleep-wake balance^1^^,^^2^^,^^6^.

The association between SC and the climacteric state is accepted, while the etiopathogenesis is not well clarified and is considered multifactorial^2^^,^^4^^,^^9^^,^^10^^,^^13^^,^^28^^-^^31^. Serum estradiol variations influence brain neurotransmitter systems, especially serotonergic ones, causing changes in sleep, mood, and memory capacity^4^. Alterations of the circadian cycle are characteristic of aging in women and may be directly related to hormonal changes^1^^,^^2^^,^^14^^,^^31^^,^^33^. On the other hand, it has been indicated that nocturnal melatonin secretion decreases with menopausal status and age^32^^,^^33^. Furthermore, exogenous melatonin in postmenopausal women has been observed to improve SC and its quality^7^.

The study found that some of the menopausal symptoms were significantly more frequent among women with SC. This has been pointed out by Shaver and Woods^2^ and Han et al.^1^, who have indicated the concomitant existence of sleep disorders with hot flashes, changes in the regularity of menstrual cycles, depressed mood, and emotional lability.

Women with moderate to severe hot flashes are approximately three times more likely to report frequent nighttime awakenings, compared to those without these symptoms^30^. The severity of sleep disorders has been reported to increase with the intensity of hot flashes^4^. Although hot flashes and their causal or associative relationship with SC and insomnia have been studied, there are not precise explanations^1^^,^^4^^,^^6^^,^^7^^,^^33^. Questions remain about the role of the hypothalamic thermoregulatory nucleus and neurobiochemical mediators with hot flashes in menopause^6^. Not all climacteric women with SC have hot flashes; therefore, other mechanisms should be considered: menopausal symptoms, mood disorders, medical conditions, socioeconomic factors, habits, or cultural patterns^6^.

Cardiac disturbances, sleep disorders, physical and mental fatigue, and muscle/joint discomfort identified with MRS, were significantly associated with SC and lost significance in the adjusted model. There are insufficient studies that explain the neuroendocrine mechanisms on how the somatic, vegetative, or psychological symptoms of menopause derived from hormonal changes in the hypothalamic-pituitary-ovarian axis are interrelated with sleep pathophysiology^9^^,^^31^.

It was found in the study that severe deterioration in the quality of life was twice associated with the presence of SC. Other authors^4^^,^^14^^,^^30^ have pointed out that sleep disorders are associated with a decrease in quality of life and with a 6-8 times greater risk of deterioration of it. The deterioration in the quality of life generated by the sleep disorders increases costs in the health system due to the greater use of medical services, absenteeism from work, risk of accidents, and lower productivity. Sleep disorders lead to poorer quality of life and decreased labor productivity and vice versa^34^. Good sleep quality is necessary for good health and quality of life.

No significant association was observed between Afro-descendants and Indigenous people with SC. Pien et al.^35^ found no association between race and quality of sleep. However, other authors^31^ found significant differences in the percentage of sleep disorders according to race/ethnicity (*p*<0.0001), similar to what was determined in the present study where among Afro-descendants it was 73.9%; Indigenous, 75.0% and mestizo, 91.5% (*p*=0.001). Overall, Caucasian women have been reported to have a higher rate of difficulty falling asleep than Hispanic women, and sleep disorders range from 28% in Japanese to 40% in Caucasians^36^.

Marital status and most work activities did not behave as factors associated with SC, similar to those announced by other authors^31^^,^^35^. Having professional activity was associated with 84.0% lower presence of SC. Baker et al.^6^ indicate that high educational level and satisfactory marriage can be protective against sleep disorders.

Alcohol, wine, or cigarette consumption was not associated with SC, although the frequency of use was very low. It has been pointed out that alcoholism behaved as a strong risk factor for insomnia in middle-aged Latin American women^4^, as well as the existence of an association between sleep quality and cigarette consumption (*p*=0.03)^35^. Woods and Mitchell^31^ reported that they observed a correlation between difficulty sleeping or falling asleep and the number of alcoholic beverages (β3: -0.023; *p*=0.046), but not with the number of cigarettes smoked (β3: -0.001; *p*=0.604). On the other hand, in a cross-sectional study in Latin American women between 40 and 59 years of age, it was pointed out that cigarette is associated with SC^13^. Additionally, the study showed that the quality of life improved with the cessation of tobacco use^37^.

In Colombia, coffee consumption is high, so it was observed in the study that 83.8% of respondents indicated that they drink it daily. Daily coffee consumption was not observed to be a factor associated with SC. Taavoni et al.^11^ also did not find it in a cohort of postmenopausal women, nor Pien et al.^35^ when evaluating coffee consumption and sleep quality. Other authors^31^ point out the opposite, as they found a correlation between difficulty sleeping and the number of drinks ingested that contain caffeine (β3: 0.020; *p*=0.006). A study of 148.938 postmenopausal women, evaluated with the Women’s Health Initiative Insomnia Rating Scale, indicated that coffee consumption was associated with less sleep disturbance^12^.

In the unadjusted and adjusted analysis, probable depression, identified by GADS, was associated with nine times greater presence of SC (*p*<0.05), it was practically the only factor identified in the study for increased SC. In a group of Latin American climacterics, also evaluated with GADS, it was found that the presence of depression increased the risk of insomnia and poor quality of sleep, OR 2.39 (95% CI: 2.10-2.72) and OR 2.48 (95% CI: 2.17-2.83), respectively^4^. Other authors^31^ also reported that the greater the severity of the symptoms of depression, the greater the difficulty in falling asleep (β3: 0.180; *p*<0.0001). Depression is a strong predictor of difficulty sleeping and poorer quality of sleep (*p*<0.0001)^35^, in this sense it should be borne in mind that the relationship between mood disorders and SC is bidirectional^6^. Most of the information regarding that negative mood induces or accentuates poor sleep quality, arises from studies where exposure to stressors: major catastrophes, divorces or mourning, amplify poor sleep quality, and a greater number of the recording of poor sleep patterns^2^. It has been proposed that SC could be considered as early markers of mood disorders^38^.

No association was found between probable anxiety with SC. An association has been reported between anxiety and more night awakenings^31^, worse sleep quality^35^, insomnia, and poor quality of sleep^4^. Family role changes, loss of loved ones, and concerns about health and aging are part of the set of stressors that women face in their personal lives, which promote states of anxiety. At the same time, stress factors can contribute to sleep disorders^6^.

A higher proportion of women in the SC group, when compared with women without SC, presented the perception of stress and loneliness. The SWAN^3^ study reported that middle-aged women with chronic exposure to stress were more likely to have insomnia than those with moderate exposure. An association among stress, sleep quality^35^, and night awakenings has been indicated^31^.

There are apparently few studies evaluating the association between loneliness and SC in the climacteric. Simon and Walker^39^, studying a few adults with an average age of twenty years, found that the absence of sleep carried a behavioral profile of social isolation and loneliness. Likewise, in a longitudinal study in 5698 older adults, evaluated with the UCLA loneliness scale and the JJS, they identified that the group with greater loneliness presented more SC; the presence of short sleep OR 1.30 (95% CI: 1.03-1.63), the difficulty in falling asleep and then getting up tired were greater^40^. The mechanisms by which the perception of loneliness exacerbates the sleep disorders, or vice versa, have not been elucidated, but a two-way relationship has been observed; the time spent awake trying to sleep fosters distress over perceived isolation and lack of sleep, hinders the person’s ability to relate to others^41^.

The study has the limitations of cross-sectional studies: it allows association and non-causality to be established. The results are specific to the population studied, not necessarily extrapolated to other communities in Colombia or Latin America, so it is necessary to carry out additional studies in other population groups. Nutritional or dietary factors, hormone levels, sleeping habits, and environments were not determined in the studied, situations that modify the SC. The results are subjective when obtained with a perception scale and there are limitations when comparing them with other studies, due to heterogeneity in the populations, tools used, and the sleep components evaluated. The JJS, a tool used to identify SC, is poorly understood and not previously validated in the Colombian population. A study^17^ in the Spanish climacteric population, with a Spanish version, had good reliability and was used in the present study. Consistency validation of JJS in various population groups is warranted. Adequate Cronbach’s alpha was obtained in this study. Polysomnography was not used as an objective tool since it was not available for economic reasons. The study has the strength to provide information on SC of climacteric women in community settings. With an adequate sample size for the selected cities, with various international scales, seeking to raise awareness of the presence of SC in climacteric women, who have not needed of consult due to changes in the sleep pattern. However, more studies in this regard are necessary.

Academic menopause and sleep science societies are encouraged to increase the exploration of SC in the climacteric. Health care entities should pay greater attention to sleep disorders since they are associated with cardiovascular pathologies, diabetes, depression, anxiety, and abuse of hypnotics that generate a significant economic burden on the health system^4^^,^^5^^,^^6^^,^^7^^,^^14^^,^^34^. Health professionals must comprehensively address climacteric and menopause, including from primary care, to identify SC and expressions of deteriorating mental health.

## CONCLUSION

Almost 17% of healthy climacteric women, approached in their homes in cities of the Colombian Caribbean, presented SC. Among the factors associated with a greater presence of SC, several menopausal symptoms and severe psychological, urogenital, and quality of life deterioration were observed. In an adjusted regression model, it was identified that probable depression was the only factor associated with SC.
